# The Effects of Air Pollution on Neurological Diseases: A Narrative Review on Causes and Mechanisms

**DOI:** 10.3390/toxics13030207

**Published:** 2025-03-13

**Authors:** Margaret Lane, Eleise Oyster, Yali Luo, Hao Wang

**Affiliations:** Department of Environmental Health Sciences, School of Public Health, University of Michigan, Ann Arbor, MI 48109, USA; marglane@umich.edu (M.L.); eoyster@umich.edu (E.O.)

**Keywords:** air pollution, neurological diseases, oxidative stress, inflammation

## Abstract

Air pollution has well-documented adverse effects on human health; however, its impact on neurological diseases remains underrecognized. The mechanisms by which various components of air pollutants contribute to neurological disorders are not yet fully understood. This review focuses on key air pollutants, including particulate matter (PM_2.5_ and PM_10_), nitrogen dioxide (NO_2_), ozone (O_3_), carbon monoxide (CO), and diesel exhaust particles (DEPs). This paper summarizes key findings on the effects of air pollution on neurological disorders, including autism spectrum disorder (ASD), attention deficit hyperactivity disorder (ADHD), Alzheimer’s disease (AD), and Parkinson’s disease (PD). Although the precise biological mechanisms remain to be fully elucidated, evidence suggests that multiple pathways are involved, including blood–brain barrier disruption, oxidative stress, inflammation, and the activation of microglia and astrocytes. This review underscores the role of environmental pollutants as significant risk factors for various neurological diseases and explores their mechanisms of action. By advancing our understanding of these interactions, this work aims to inform new insights for mitigating the adverse effects of air pollution on neurological diseases, ultimately contributing to the establishment of a cleaner and healthier environment for future generations.

## 1. Introduction

The detrimental effects of air pollution on human health have long been a subject of concern, with its insidious impacts continuing to afflict the modern world. The harmful consequences of air pollution were first recognized during the Industrial Revolution in the 18th century, a period characterized by significant technological advancements that led to a dramatic increase in the release of pollutants such as sulfur dioxide (SO_2_) and particulate matter (PM) into the atmosphere. These pollutants were associated with an increased risk of chronic respiratory diseases and, in extreme cases, mass mortality events [1]. While early research primarily focused on the adverse effects of air pollution on respiratory and cardiovascular outcomes, a growing body of recent studies suggests that its detrimental effects extend to the central nervous system (CNS), potentially accelerating neurodegenerative processes. As societal advancements continue, marked by increased vehicle usage and industrial activity, pollutant emissions into the air have risen substantially, underscoring the urgent need to investigate the role of air pollution in the development of neurodegenerative neurological diseases.

This review focuses on the role that air pollution plays in neurological diseases, with a specific focus on autism spectrum disorder (ASD), attention deficit hyperactivity disorder (ADHD), Alzheimer’s disease (AD), and Parkinson’s disease (PD). ASD is a neurodevelopmental disorder characterized by deficits in social behavior and communication, repetitive behaviors, and stereotyped behaviors [2,3]. Similarly, ADHD, another neurodevelopmental disorder, is characterized by inattention, impulsivity, hyperactivity, and learning difficulties [4]. In addition, AD, the leading cause of dementia, is characterized by progressive neuronal degeneration that impairs memory, cognition, and behavior [5]. Furthermore, PD, a progressive neurodegenerative disorder, is primarily characterized by motor symptoms such as tremors, rigidity, and bradykinesia, often accompanied by cognitive decline, psychiatric complications, and a significant reduction in quality of life [6]. This review places particular emphasis on key pollutants, including particulate matter (PM_2.5_ and PM_10_), nitrogen dioxide (NO_2_), ozone (O_3_), carbon monoxide (CO), and diesel exhaust particles (DEPs).

In recent years, the hypothesis that air pollution contributes to neurological system damage has gained significant attention. A growing body of research has established a strong foundation for the idea that air pollution is a critical factor in significant neurological disease-related outcomes (Table S1). Human and animal studies have established a robust positive correlation between increased exposure to environmental pollutants and a higher risk of developing ASD [7,8,9,10]. Current research also highlights the significant role of pollutants such as PM, NO_2_, and DEPs in the pathogenesis of ASD [9,10,11,12]. Similarly, emerging studies suggest that air pollutants are an important factor in the development of ADHD [13,14,15], with strong associations observed between pollutants like PM_2.5_, NO_2_, and CO and the development of ADHD [16,17]. In addition, related reviews and analyses indicate a strong association between air pollution and neurodegenerative diseases like AD and PD [5,18,19]. Studies also suggest that air pollution exacerbates cognitive decline and disease progression in AD and PD, further underscoring the need for public health initiatives to mitigate air pollution [20,21,22]. Both human and animal studies provide compelling information on the potential mechanisms, including neuroinflammation and amyloid plaque deposition, providing new insights for the further investigation of the neurotoxicity of air pollution [23,24,25].

Although these neurological diseases manifest differently, their underlying mechanisms of detrimental effects share common features. For instance, ASD and ADHD are polygenic disorders influenced by multiple risk factors rather than a single gene [26]. While genetic factors are contributors to AD and PD, accounting for approximately 10% of cases, the majority of cases are sporadic [27]. A key similarity between all of these disorders is the role of oxidative stress. A substantial body of research indicates that oxidative stress and neuroinflammation contribute to the etiopathology of different neurological disorders [3]. Furthermore, research has emphasized that neuronal changes, such as neuroinflammation, are related to behavioral alterations observed in all of these disorders [28].

While the detrimental effects of air pollution on the respiratory and cardiovascular systems have long been recognized, the knowledge about the effects of air pollution on neurological diseases is still very limited. This review aims to identify the specific air pollutants and chemicals that are strongly associated with neurological diseases, summarize the current knowledge on their mechanisms of action, and highlight their association with neuropathological changes. By addressing critical gaps in current research, this review seeks to inform public health strategies and underscore the importance of air pollution as a key risk factor for the increasing global burden of neurological diseases.

## 2. Methodology

The methodology for this review involved a comprehensive literature search of original research or substantial reviews from 2006 to 2024. This review includes studies published in peer-reviewed journals that focus on the relationship between air pollution and its impact on neurodevelopmental or neurodegenerative disorders. A combination of keywords related to neurotoxicity, including “neurotoxicity”, “air pollution”, “NO_2_”, “CO_2_”, “PM_2.5_”, “O_3_”, “oxidative stress”, “autism”, “Alzheimer’s disease”, and “Parkinson’s disease”, was used to find the literature referenced in this review. These keywords captured a broad range of epidemiological and animal model studies examining the effects of environmental toxins on neurological health. The search was conducted across several electronic databases, including PubMed, ScienceDirect, JSTOR, and Google Scholar. The aforementioned databases were chosen for this review due to their comprehensive coverage of the biomedical and environmental science literature. Conversely, we excluded studies that involved non-environmental neurotoxicity-related factors or lacked relevant data. We further refined this careful selection process by reviewing the reference lists of key articles to identify additional relevant literature.

## 3. Air Pollutants in the Development of Neurological Disease

Air pollution comprises a complex mixture of particulate matter (PM_2.5_ and PM_10_), nitrogen oxide (NO), nitrogen dioxide (NO_2_), carbon monoxide (CO), sulfur dioxide (SO_2_), ozone (O_3_), and volatile organic compounds (VOCs) and has become one of the most important environmental issues in the world. Its impact on human health is an escalating concern, with pollutants such as PM_2.5_, PM_10_, NO_2_, and O_3_ being widely recognized as significant environmental risk factors for different neurological diseases [29]. It should also be considered that the brain consumes the most oxygen of any organ within the body and therefore is going to be acutely influenced by any disturbance to the blood–brain oxygen transfer. Current research focuses on several key air pollutants, including PM_2.5_ and PM_10_, NO_2_, O_3_, CO, and DEPs. While motor vehicle emissions are a major source of these pollutants, industrial boilers and factory emissions, as well as natural events such as volcanic eruptions, wildfires, and dust storms, also contribute significantly to their presence [3]. As evidence of their neurotoxicological effects continues to accumulate, understanding the mechanisms through which these pollutants affect human health becomes increasingly critical [30,31].

### 3.1. Particulate Matter

Particulate matter, especially PM_2.5_ and PM_10_, is recognized as one of the most toxic air pollutants, with PM_2.5_ posing a greater threat due to its smaller size. The greatest sources of PM include motor vehicles’ fuel combustion, industrial facilities’ combustion processes, wildfires, and brush burnings. PM_2.5_ refers to airborne particles with a diameter of less than 2.5 μm, enabling them to easily penetrate biological systems [9,29]. Due to its diminutive size, PM_2.5_ can reach the alveoli in the lungs and even travel to the brain through systemic circulation or the olfactory bulb [29]. The toxicological profile of PM_2.5_ includes various toxic components such as polycyclic aromatic hydrocarbons (PAHs), heavy metals, organic and inorganic compounds, and reactive gases, all of which can induce oxidative stress and neuroinflammation [9,32]. A previous study found that PM_2.5_ exposure decreased the survival rate of neuron cells and disrupted mitochondrial morphology while decreasing ATP levels as well as decreasing the mRNA and protein expression levels of survival genes (CRB and Bcl-2) and neuroprotective genes (PPARү and AMPK) and significantly increased the levels of oxidative stress and the expression of inflammatory mediators in SH-SY5Y neuronal cells (TNF-ɑ, IL-1β, and NF-κB) [33]. Along with gaining access to the brain via blood and olfactory pathways, it has long been believed that these pollutants once inhaled do damage to the lungs and can influence neurological disorders via hypoxia-induced damage and inflammatory responses [2,3,9]. These characteristics come together to indicate that PM_2.5_ is highly detrimental to the CNS and has extreme neurotoxic effects [33].

### 3.2. Nitrogen Dioxide

Nitrogen dioxide (NO_2_) is a harmful, highly reactive gas and serves as a standard indicator for nitrogen oxides (NO_x_) as a group [34]. NO_2_ is primarily released into the atmosphere through fuel combustion, with significant contributions from the emissions of vehicles, power plants, and off-road machinery [34]. Indoor sources of NO_2_ include the burning of fuels like wood and gas [34]. Extensive evidence has linked NO_2_ exposure to adverse health effects, making it one of the six major pollutants regulated by national air quality standards [15]. The acute inhalation of NO_2_ induces oxidative stress, which leads to the generation of reactive oxygen species (ROS) that damage brain cells [35]. This oxidative stress, combined with mitochondrial dysfunction, impairs energy metabolism, reduces ATP production, and disrupts mitochondrial biogenesis, all of which are crucial for proper neuronal function [35]. Mitochondrial damage resulting from NO_2_ exposure can contribute to neuronal injury and has been associated with an increased risk of cognitive deficits, ischemic stroke, and neurodevelopmental impairments [35]. Despite a significant decrease in NO_2_ emissions in the U.S., from 15 million short tons in 2011 to approximately 7.64 million short tons in 2020, NO_2_ remains an important environmental health concern. Furthermore, NO_2_, in combination with other NO_x_, interacts with volatile organic compounds (VOCs) and other chemicals in the atmosphere to form PM and O_3_ [36]. Sunlight-driven photochemical reactions catalyze these processes, freeing oxygen atoms to react with other atmospheric compounds, ultimately forming ground-level ozone [36].

### 3.3. Ozone

Ozone (O_3_) naturally exists in the stratosphere, forming a protective layer that shields the Earth from harmful ultraviolet radiation. However, ground-level ozone in the troposphere, generated by human activities, poses significant health risks to humans [37]. O_3_, a highly reactive oxidant, is one of the most prevalent urban air pollutants. Over 30% of the U.S. population lives in areas with unhealthy O_3_ levels. Certain occupations, such as those in pulp mills and outdoor construction, involve intermittent exposure to relatively high O_3_ concentrations (0.3–1 ppm) in the workplace [38,39]. O_3_ exposure can induce the constriction of airway muscles, trapping air in the alveoli and causing respiratory symptoms such as wheezing and shortness of breath [5]. Although primarily targeting the lungs, O_3_ can also enter the bloodstream and reach the brain via the respiratory or olfactory route, inducing neurotoxic effects [5]. High levels of O_3_ exposure have been implicated as a potential risk factor for neurological conditions [40]. Research indicates that O_3_ exposure can trigger inflammatory responses in the brain, characterized by elevated levels of proinflammatory cytokines and activated microglia, which are critical components in the pathogenesis of neurological diseases, particularly Alzheimer’s disease [5]. Moreover, long-term exposure to ambient O_3_ has been linked to cognitive decline, especially among older adults [41]. It has been observed that individuals exposed to higher levels of O_3_ experience impairments in memory and executive functioning, increasing the risk of dementia-related disorders [41].

### 3.4. Carbon Monoxide

Carbon monoxide (CO) is an odorless, inorganic gas produced by the incomplete combustion of carbon-based fuels, with primary sources including vehicle exhaust and tobacco smoke. Gas stoves are another significant source of indoor CO [2]. Once inhaled, CO rapidly diffuses across the alveolar–capillary membrane and binds to hemoglobin with a much greater affinity than oxygen, forming carboxyhemoglobin [2]. This disrupts oxygen delivery to tissues, which may lead to hypoxic conditions and impair oxidative phosphorylation [2]. Toxic concentrations of CO induce mitochondrial depolarization, the inhibition of nicotinamide adenine dinucleotide + hydrogen (NADH)-dependent respiration, and the generation of ROS, which results in lipid peroxidation and a decrease in glutathione [42]. The inhibition of these phases creates CO-induced oxidative stress and cell death in neurons and astrocytes [42]. Due to its colorless and odorless nature, CO exposure frequently goes unnoticed, making its poisoning the leading cause of poison-related mortalities in the U.S. [2]. The National Institutes of Health (NIH) estimates that approximately 50,000 individuals in the U.S. suffer from CO poisoning annually [43].

### 3.5. Diesel Exhaust Particles

Diesel exhaust particles (DEPs) are another major air pollutant, containing fine particulate matter along with elemental carbon and absorbed organic compounds such as PAHs and nitrated PAHs. Trace elements like sulfate, nitrates, and metals are also present in DEPs [44]. Diesel emissions contribute significantly to air pollution, accounting for up to 28% of PM pollutants. The fine particles in diesel exhaust, with a core diameter of 10–30 nm, can penetrate deep into the respiratory tract. The slow clearance mechanisms in the respiratory epithelium allow prolonged interaction with tissues, increasing the chance of particles entering the bloodstream and translocation to other tissues. Research indicates that exposure to DEPs can lead to oxidative damage, neuroinflammation, and microglial activation, all of which are processes implicated in the pathogenesis of neurological diseases [45]. The same study also shows that high concentrations of DEPs can cause structural changes in brain tissue, such as white matter injury and alterations in amyloid-β peptide levels [45]. Due to its composition, diesel exhaust is classified as both mutagenic and carcinogenic to humans by the WHO. In addition, the NIH identifies it as among the most prevalent anthropogenic pollutants globally.

**Figure 1:**
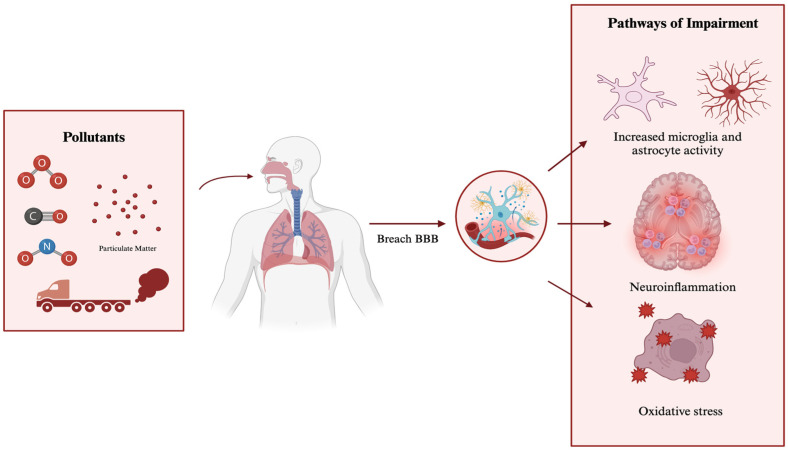
Possible pathways of air pollutants in neurological disorders.

The harmful effects of these pollutants extend beyond the respiratory and cardiovascular systems, impacting both systemic health and brain functions through various translocation pathways (Figure 1). During inhalation, some particles are ingested into the throat and absorbed into the olfactory epithelium [3]. From there, particles can then travel to the olfactory bulb and spread to the olfactory cortex and other regions of the brain [3]. In addition, it is suggested that PM, in particular, can enter the brain directly through the olfactory nerve, bypassing the blood–brain barrier (BBB) [3]. As previously discussed, these air pollutants are primarily deposited in the lungs, from where they can translocate through the blood to the brain and other organs. Although this pathway is not the most efficient, it accounts for some of the presence of toxicants within organs [3]. During this process, toxic particles can trigger various reactions in cells, including microglial activation, oxidative stress, and neuroinflammation [3]. These biological responses are strongly associated with various types of neurological disorders and diseases [3].

## 4. Association Between Air Pollution and Neurological Diseases

### 4.1. Autism Spectrum Disorder

Autism spectrum disorder (ASD) is a neurodevelopmental disorder characterized by deficits in social interaction and communication, as well as restrictive and repetitive behaviors [2]. Research has found that ASD typically manifests before the age of three and is often associated with impairments in cognitive function, learning, attention, and sensory processing [3]. Over recent years, the prevalence of ASD has been steadily increasing, which may be partly attributed to enhanced awareness, improved diagnostic methods, and evolving definitions of the disease [3,9]. However, the etiology of ASD is still very limited. Current findings suggest that ASD is not linked to a singular gene but rather arises from complex interactions between multiple genes [46]. In fact, it was reported that hundreds of genes have been identified to converge on selective biological domains to be either causative or risk factors of ASD [47]. In addition to genetic predisposition, environmental factors, including air pollution, may significantly contribute to its development [2]. This hypothesis is further supported by epidemiological studies identifying air pollution as a risk for the development of ASD in humans [3,10].

#### 4.1.1. Epidemiological Evidence

Many epidemiological studies have established a link between air pollutants and the development of ASD. Studies conducted in the United States, Taiwan, and Israel have demonstrated that exposure to air pollution components such as PM_2.5_, NO, and NO_2_ is positively associated with an increased risk of ASD [48,49,50]. A study examining proximity to freeways and major roads, using maternal addresses from birth certificates and residential histories obtained through questionnaires, found that gestational and early-life exposure to traffic-related air pollution was associated with an increased risk of ASD [11]. Dutheil et al. (2021) analyzed previous studies on ASD risk in children and found that PM_2.5_ presented a greater risk of ASD compared to PM_10_, NO_x_, and other pollutants [9]. Notably, all studies focusing on maternal exposure to PM_2.5_ consistently reported an elevated risk of ASD in newborns [9]. The primary components of PM_2.5_ include elemental carbon/black carbon (EC/BC), nitrate (NO_3_^−^), sulfate (SO_4_^2−^), and organic matter (OM) [51]. A retrospective cohort study also found that prenatal exposure to major PM_2.5_ components, including EC/BC, OM, and, to a lesser extent, SO_4_^2−^, was associated with an increased risk of ASD [52]. Accumulating evidence suggests that exposure to PM_2.5_ can adversely affect neurodevelopment, potentially contributing to ASD in human studies. For instance, long-term exposure to PM_2.5_ over the preceding 5–20 years has been linked to a reduction in total deep-gray brain volumes, suggesting cumulative brain damage and atrophy [53]. Additionally, elderly women living in areas with high levels of PM_2.5_ exhibited decreased white matter volume in the corpus callosum and frontal and temporal lobes [54]. PM_2.5_ has also been shown to penetrate various biological barriers, including the blood–alveolar barrier, placental barrier, and BBB, leading to inflammation and oxidative stress in the brain [55]. Given that hippocampal impairment is a hallmark of ASD, these findings provide evidence linking prolonged PM_2.5_ exposure to deficits in learning, memory, and emotional regulation commonly associated with ASD [56]. Furthermore, individuals with ASD exhibit higher levels of oxidative stress, along with increased neuro- and systemic inflammation, which play significant roles in modulating neurodevelopmental disorders, including ASD [3]. Air pollution has been shown to induce such inflammation, as demonstrated by an autopsy study that found elevated cerebral neuroinflammation in residents of high-pollution areas compared to those in low-pollution areas [15]. Given these findings, it is crucial to explore the explicit links between air pollution and ASD through controlled and causal experimental designs utilizing animal models.

#### 4.1.2. Animal Studies

Animal models are invaluable tools in research, and studies using animal models have shown that air pollution contributes to the development of ASD. For example, developmental exposure to diesel exhaust in mice from embryonic day 0 to postnatal day 21 resulted in reduced social interaction in social preference tests and reciprocal interaction, increased repetitive behaviors in the marble-burying tests and T-maze, and impaired communication assessed by measuring responses to social odors and isolation-induced ultrasonic vocalizations [7]. The brain is particularly vulnerable to oxidative stress, and maternal exposure to PM_2.5_ during pregnancy has been shown to cause pathological changes in the cerebral cortex, including a reduced number and diameter of neurons, as well as increased neuronal apoptosis in offspring mice [57]. High-dose PM_2.5_ exposure could reduce the number of presynaptic vesicles in offspring mice, indicating detrimental effects on synaptic plasticity [57]. In addition, long-term PM_2.5_ can significantly reduce apical spine density in the cornu ammonis 1 (CA1) region of the hippocampus, impaired dendritic complexity, and decreased apical dendritic length in the pyramidal neurons of the CA3 region [58]. Another study found that exposure to ambient ultrafine particles induced inflammation and microglial activation, reduced the size of the corpus callosum and hypomyelination, and disrupted white matter development and ventriculomegaly in mice, accompanied by increased astrocytic activation in the amygdala, elevated glutamate levels, and excitatory/inhibitory imbalance [59]. Prenatal and nursing exposure to DEPs affected CNS development by increasing locomotor activity, self-grooming behaviors in the presence of unfamiliar mice, and rearing behaviors, mirroring the restrictive and repetitive behaviors seen in ASD patients [10]. Collectively, these findings suggest that air pollution may be an important risk factor for the development of ASD.

#### 4.1.3. Biological Mechanisms

Studies have also investigated the potential mechanisms by which air pollution contributes to ASD. Neuroinflammation is widely acknowledged as a significant risk factor for ASD, which can disrupt brain functions through several mechanisms, including the inhibition of neurogenesis, hypomyelination, and the reduced expression of reelin, a signaling glycoprotein essential for neuronal migration and polarity [3]. During pregnancy, inhaled air pollutants such as PM can trigger systemic inflammatory responses in the fetus, leading to neuroinflammation in the developing brain. It must be considered that during fetal development and early childhood there are periods of rapid cellular differentiation and growth, putting them at acute risk of damage from environmental pollutants [15]. In addition, air pollution encroaching on a mother’s oxygen supply is detrimental, as a placenta under stress from a lack of oxygen can compromise the differentiation and organization of the fetal brain, which is thought to influence ASD development [9,52]. Due to the immaturity of the BBB, PM can directly translocate to the fetal brain, activating inflammation in astrocytes and microglia [60]. This process releases proinflammatory cytokines and activates key inflammatory pathways, such as NF-κB and JNK [61,62]. A previous study indicated that PM can activate TLR-4 on the microglial membrane, initiating a cascade of signal transduction that phosphorylates the downstream nuclear transcription factor NF-κB, resulting in the increased expression of proinflammatory cytokines (TNF-α, IL-6, and IL-1β) and microglia pathogenicity [63]. The mitogen-activated protein kinase (MAPK) signaling pathway is also highly sensitive to air pollutants. PM_2.5_ exposure can induce the phosphorylation of p38 MAPK, a component of the MAPK pathway and a critical mediator of inflammation [64]. In the CNS, MAPK activation in microglia and astrocytes promotes the secretion of proinflammatory cytokines, contributing to neuroinflammation and potentially disrupting synaptic plasticity in regions associated with ASD, such as the prefrontal cortex [61]. Additionally, these stresses can disrupt the balance between excitatory (glutamate) and inhibitory (gamma-aminobutyric acid [GABA]) neurotransmitter systems, a hallmark feature of ASD [65]. Inflammatory responses in astrocytes and microglia can disrupt normal neural development by inducing neuronal apoptosis, reducing neuronal density, and impairing synapse formation and maturation, further impairing oligodendrocytes and disrupting white matter myelination, resulting in reduced white matter volume [66,67]. These consequences have been shown to contribute to the pathogenesis and progression of ASD.

The brain is uniquely vulnerable to excessive oxidative stress, with microglia and astrocytes serving as primary regulators by generating reactive oxygen species (ROS) and modulating antioxidant defense pathways [68]. Exposure to air pollution has been shown to elevate oxidative stress, particularly lipid peroxidation products like malondialdehyde (MDA) and thiobarbituric acid reactive substances (TBARs), as well as hydrogen peroxide (H_2_O_2_) [69,70,71]. Key antioxidant proteins, including glutathione (GSH), nuclear factor erythroid 2-related factor 2 (Nrf2), heme oxygenase 1 (HO-1), superoxide dismutase (SOD), and catalase (CAT), are often elevated, indicating increased detoxification capacities [72,73]. However, decreases in these proteins in some cases suggest the potential impairment of the detoxification machinery [74,75,76]. Oxidative stress is a major contributor to neuroinflammation and the disruption of astrocyte–neuron crosstalk, ultimately leading to ASD [68]. Studies have suggested that oxidative stress caused by air pollution is associated with alterations in neurotransmitter levels [77]. Air pollutants can reduce dopamine and serotonin levels, key neurotransmitters involved in reward processing and motor function, in the striatum, leading to functional impairments characteristic of ASD [78]. Furthermore, air pollution exposure has been linked to reductions in dopamine and norepinephrine in the prefrontal cortex, potentially impairing executive function and decision-making abilities [79].

### 4.2. Attention Deficit Hyperactivity Disorder

Attention deficit hyperactivity disorder (ADHD) is another neurodevelopmental disorder that typically manifests in childhood and can persist into adulthood. Individuals with ADHD exhibit symptoms of inattention, hyperactivity, and impulsivity, often accompanied by behavioral issues, emotional dysregulation, and learning difficulties [4]. As of 2020, the global prevalence of ADHD among children and adolescents was estimated at 5% [15]. By 2024, this figure had increased to approximately 8%, according to a recent review [80]. Similar to ASD, the exact etiology of ADHD remains unclear, though it is believed to involve a combination of genetic and environmental factors. Emerging evidence suggests that environmental factors, such as air pollution, may interact with genetic predispositions to increase the risk of neurodevelopmental disorders [15]. As the rates of ADHD continue to increase, so too does the amount of research investigating the role of air pollution in this disorder.

#### 4.2.1. Epidemiological Evidence

A study focusing on CO reported that children exposed to this pollutant through tobacco smoke, either pre- or postnatally, often exhibited conduct disorders and ADHD later in life [16]. It is important to note that fetal development and early childhood are periods of rapid cellular differentiation and growth, making them particularly vulnerable to damage from environmental pollutants [15]. ADHD was identified in 11.0% of urban children exposed to air pollution, particularly particulate matter (PM_10_), compared to 2.7% in the control group [14]. Infants in the highest tertile of exposure to PM_10_ or NO_2_ had a 2- to 3-fold higher risk of developing ADHD compared to those in the lowest tertile [13]. Similarly, research also reported that children exposed to the highest quintile of NO_2_ and PM_2.5_ during childhood had a 1.70-fold and 1.63-fold, respectively, increased risk of ADHD when compared to those exposed to the lowest quintiles of these pollutants [15]. It has also been suggested that increasing exposure to N_2_O from air pollution may be a significant contributor to ADHD [81]. Environmentally relevant concentrations of N_2_O, even in trace amounts, have been shown to impair cognitive functions, such as working memory in adult human males [82]. Furthermore, exposure to PAHs, particularly bisphenol A (BPA), has been associated with ADHD symptoms in primary school children. These toxicants are significantly associated with a reduction in the volume of the caudate nucleus, a brain region critical for cognitive and behavioral processes [83,84]. Collectively, these findings suggest that early-life exposure to air pollutants can significantly increase the risk of ADHD [15].

#### 4.2.2. Biological Mechanisms

Research efforts have also been made to elucidate the mechanisms underlying the effects of air pollution on ADHD. PM, along with associated organic compounds (e.g., PAHs) and metals (e.g., Pb, As, and Mn), may initiate molecular and cellular events that contribute to ADHD. PM exposure can also directly or indirectly induce oxidative stress and inflammation, both of which are implicated in the etiology of ADHD [85]. PM exposure can disrupt thyroid hormone signaling, leading to hypothyroidism, reduced brain-derived neurotrophic factor (BDNF) levels, and the dysregulation of GABAergic interneuron function [86,87]. These disruptions can impair synaptogenesis and induce neural network dysfunction [88]. Additionally, PM may interfere with β-adrenergic, dopamine, and glutamate (NMDAR) signaling pathways, causing a disturbance in G-protein/cAMP signaling, Ca^2+^ homeostasis, and neurotransmitter pathways [89,90]. Several studies have demonstrated that prenatal exposure to PM in mice induced the dysregulation of dopamine and its metabolites in various brain regions [91,92]. β2-adrenergic receptors (β2ARs) mediate physiological responses to noradrenaline and play a critical role in normal neurodevelopmental processes during fetal development [93]. Recent studies have revealed that benzo[a]pyrene (B[a]P) and PAHs can directly bind to β2AR, activating downstream signaling pathways in vitro, subsequently leading to β2AR desensitization [94,95]. Furthermore, the PAH-induced disruption of β2AR signaling, in conjunction with aryl hydrocarbon receptor (AhR) signaling, has been proposed as a key mechanism underlying neurotoxicity [96]. PAHs may bind to AhR, altering the gene regulation of NMDAR subunits and disrupting Ca^2+^ homeostasis, thereby compromising BDNF signaling [97]. In addition, N_2_O exposure has been shown to reversibly inhibit human alpha-7 nicotinic acetylcholine receptors (α7 nAChRs), a neuronal receptor subtype that regulates synaptic neurotransmitter release in the CNS [98]. This receptor is essential in enhancing the glutamatergic activity that supports working memory and attention-related pathways in the prefrontal cortex [99]. The inhibition of α7 nAChR by N_2_O exposure may lead to oxidative stress, characterized by increased levels of peripheral NO [100]. Since NO promotes parasympathetic activity, excessive NO levels could drive a parasympathetic-dominant state in ADHD [101]. Supporting this, α7 nAChR activation on sympathetic nerves facilitates norepinephrine (NE) release, leading to neuronal vasodilation [102]. Thus, the N_2_O-mediated inhibition of α7 nAChR may impair sympathetic activity and contribute to the etiopathology of ADHD. Taken together, these mechanisms may collectively contribute to the adverse outcomes associated with air pollutant exposure, ultimately increasing the risk of ADHD.

### 4.3. Alzheimer’s Disease

Alzheimer’s disease (AD) is a leading cause of dementia among individuals aged 65 and older. It affects approximately 50 million people worldwide, with projections indicating that this number will rise to 135 million by 2050 [5]. As a significant global health concern with substantial social and economic burdens, AD remains a priority for research and intervention. The main risk factors of the disease include age, diet, genetics, and environmental factors [5]. Systematic reviews and meta-analyses indicate that air pollution is involved in AD development.

#### 4.3.1. Epidemiological Evidence

In 2020, Fu and Yung conducted a comprehensive meta-analysis of 22 studies, revealing that exposure to PM_2.5_, NO_2_, and O_3_ significantly increases the risk of AD. Notably, PM_2.5_ exposure was associated with a 94% increase in the odds of developing AD [5]. Moreover, another study found that a 10 ppb increase in annual O_3_ levels has been associated with a cognitive decline equivalent to 3.5–5.3 years of aging [103]. In a cohort study involving 95,690 participants aged 65 and older in Taiwan, researchers observed a 211% increase in AD risk for every 10.91 ppb rise in ambient O_3_ concentrations and a 138% increase in AD risk per 4.34 μg/m^3^ rise in PM_2.5_ levels between 2000 and 2010 [104]. These findings underscore the significant role of PM_2.5_ and O_3_ in the neurodegenerative process. Similarly, Peters et al. (2019) found that PM_2.5_ and NO_2_ exposures are strongly associated with an increased risk of all-cause dementia, including AD [19]. Tsai (2019) reported that each 10 μg/m^3^ increase in PM_2.5_ corresponds to a pooled hazard ratio of 3.26 for dementia and 4.82 for AD in individuals aged 50 and older [105]. These studies suggest that air pollution is not only a risk factor for neurological disease but also accelerates cognitive decline, making it a critical public health issue.

Human studies also provide robust evidence for the toxic effects of long-term air pollution exposure on cognition. PM_2.5_ may contribute to AD development by translocating to cortical regions where the disease originates [106]. A study by Shi et al. (2023) identified consistent associations between specific PM_2.5_ constituents, including black carbon (BC), sulfate (SO_4_^2−^), and organic matter (OM), and elevated AD incidence. Specifically, an interquartile range increase in PM_2.5_ mass was associated with a 9% increase in AD incidence [107]. Among the PM_2.5_ constituents, BC exhibited the strongest effect on AD risk, with a hazard ratio for AD increase of 23–39% per 1 μg/m^3^ increase in BC levels, as modeled using a chemical-transport-based simulation model [107]. In addition, environmental nanoparticles have been found to reach noradrenergic and dopaminergic nuclei, as well as the cerebellum, causing neurovascular damage in children and young adults with neural quadruple misfolded protein pathologies in Mexico City [108].

While many studies focus on PM_2.5_, other pollutants like O_3_ also demonstrated significant neurotoxic effects. Cleary et al. (2018) found that increased ground-level O_3_, but not PM_2.5_, is directly linked to a higher rate of cognitive decline among Alzheimer’s disease study participants [109]. Multivariate Cox proportional hazards regression analysis revealed that elevated exposure to NO_2_, SO_2_, CO, and PM_10_ was associated with an increased risk of cognitive decline in AD, with SO_2_ exposure exerting the greatest impact, while O_3_ showed a neutral effect on AD-related cognitive deterioration in polluted cities [110].

#### 4.3.2. Animal Studies

Animal studies have further elucidated the potential mechanisms underlying air pollution in AD. Chuang et al. (2020) demonstrated that low-level PM_2.5_ inhalation for 3 to 6 months increases neuroinflammation and synaptic alterations, which could contribute to AD [23]. Particulate air pollution, including iron-rich nanoparticles from combustion and friction, disrupts brain chromatin silencing and compromises DNA integrity, potentially increasing AD risk, particularly in young populations [111]. Recently, Israel et al. (2023) reported that mice exposed to PM_2.5_ exhibited accelerated Alzheimer’s disease-related pathology through increased amyloid plaque deposition and oxidative stress [112]. In addition, multiple studies have consistently reported that air pollution can contribute to AD development in animal models through mechanisms such as microglia activation and the increased production of proinflammatory cytokines [32,113]. Short-term exposure to O_3_ concentrations ranging from 0.2 to 1 ppm (4 h) has been shown to disrupt long-term memory, induce brain lipid peroxidation, and reduce dendritic spine density and motor activity in young rats [114]. Additionally, O_3_ exposure impaired mitochondrial function, stimulated Aβ42 production, and reduced brain repair capacity. However, treatment with the antioxidant vitamin E mitigated O_3_-induced lipid peroxidation in the hippocampus and improved memory deficits in rats [115,116], implicating oxidative stress as the primary driver of O_3_-induced memory impairment. This coherence, biological plausibility, and consistency in the literature suggest that air pollution is a neurotoxic agent, with chronic exposure leading to cumulative damage in vulnerable brain regions.

Moreover, several studies suggest that the effects of air pollution on AD may vary based on genetic predisposition and gender. For example, nano-sized refractive particles were identified in the hippocampus of animals exposed to traffic-related air pollution (TRAP), leading to elevated levels of hyperphosphorylated tau, increased amyloid plaque deposition, significant cognitive deficits, greater neuronal cell loss, and heightened microglial activation in wildtype and genetically susceptible rats [6]. This study focused on TgF344-AD rats, which are genetically susceptible to AD due to their hemizygous expression of two human transgenes, APPswe and PSEN1ΔE9 [6]. Specifically, TRAP-exposed TgF344-AD males and females demonstrated higher levels of amyloid plaques and hyperphosphorylated tau than their WT counterparts, indicating a significant increase in neurotoxicity linked to the genetic predisposition of the TgF344-AD rats, compared with the more resilient and less affected WT rats [6].

#### 4.3.3. Biological Mechanisms

Understanding the mechanisms behind AD is essential for addressing this growing health crisis. AD is characterized by the progressive misfolding of proteins and the formation of plaques, which initially develop in specific brain regions before spreading to the entire cortex [117]. Amyloid plaque deposition is characterized by the accumulation of beta-amyloid peptides in the brain’s extracellular space [118]. These peptides, derived from the cleavage of amyloid precursor protein (APP) by enzymes, aggregate into insoluble fibrils that disrupt neuronal communication and induce cellular stress, triggering neurodegenerative processes [118,119]. The inhalation of diesel engine exhaust (DEE), a major source of particulate air pollution, has been shown to accelerate Aβ plaque formation in the cortex and hippocampus, increase whole-brain homogenate Aβ42 levels, and impair motor functions in 5XFAD transgenic mice, exacerbating the progression of AD [120]. Motor deficits are associated with spinal cord pathology, evidenced by significant intraneuronal Aβ accumulation and extracellular plaque deposition [121]. Oxidative stress and inflammation, induced by the translocation of ultrafine carbonaceous particles from DEE into the brain, are proposed as primary or complementary AD mechanisms [122]. Excessive ROS may arise from mitochondrial dysfunction and the abnormal accumulation of transition metals [123]. Neuroinflammation is attributed to the activation of microglial cells and the release of various cytokines [124]. For example, elevated levels of IL-1 have been shown to increase APP production and Aβ accumulation [125]. Additionally, the aberrant accumulation of Aβ and tau proteins can exacerbate redox imbalance and inflammation [126,127]. Evidence further suggests that neuroinflammation and oxidative stress enhance the production and aggregation of Aβ, as well as the phosphorylation and polymerization of tau, creating a vicious cycle that drives the progression of AD [128]. Further findings revealed that O_3_ exposure could induce oxidative stress, leading to endoplasmic reticulum (ER) stress in the hippocampus [129]. This study also found that ER stress could activate transcription factor 6 (ATF6), increasing glucose-regulated protein 78 (GRP78) levels in the cytoplasm and enhancing the nuclear translocation of ATF6. This nuclear translocation establishes a vicious cycle that, together with the activation of apoptotic cell death pathways, perpetuates ER stress, contributing to neurodegeneration processes like AD. In the future, more studies will be essential to fully understand these underlying mechanisms, as such insights are crucial for elucidating the relationship between air pollution and neurodegeneration.

### 4.4. Parkinson’s Disease

Parkinson’s disease (PD) is a neurodegenerative disorder clinically characterized by muscle rigidity, resting tremor, bradykinesia, and postural instability [130]. It is the second most common neurodegenerative disorder, affecting over 10 million individuals globally, with a prevalence of 1% among people over 60 years of age (National Institutes of Health [NIH], 2022). Beyond its clinical manifestations, PD imposes a significant economic burden, estimated at USD 51.9 billion annually in the United States [99]. Given its substantial public health and economic impact, a growing body of research has focused on identifying the environmental factors that may contribute to its development.

#### 4.4.1. Epidemiological Evidence

Many epidemiological studies have shown an association between PD and air pollutants, including PM_2.5_, O_3_, and NO_2_ [18]. For example, ambient air pollution has been linked to a 9% increased risk of PD per interquartile range increase (2.97 μg/m^3^) in modeled NO_2_ levels [131]. A case–control study using a hierarchical Bayesian model to estimate four-year ambient PM_2.5_ levels demonstrated a positive association between PM_2.5_ exposure and PD risk [20]. Notably, subgroup analyses revealed that PM_2.5_ and PM_10_ exposure could increase PD risk, particularly among female never-smokers [132]. In addition, PM_2.5_ and NO_2_ exposure has been significantly associated with an increased risk of developing dyskinesia and the akinetic–rigid PD subtype [133]. Furthermore, a study in North Carolina found a significant positive association between PD and both O_3_ (odds ratio = 1.39; 95% CI: 0.98 to 1.98) and PM (odds ratio = 1.34; 95% CI: 0.93 to 1.93) [20]. An epidemiological investigation revealed that residents exposed to higher PM_2.5_-Mn concentrations (203 ng/m^3^) exhibited worse motor function, slower finger-tapping velocities, and longer completion times on the grooved pegboard test, which are linked to clinical parkinsonism [21]. While meta-analyses consolidate evidence from diverse sources, highlighting that environmental pollution levels significantly modulate the risk of neurodegenerative diseases, genetic factors also play a critical role in modulating PD risk. Nalls et al. (2019) provided complementary insights by identifying novel genetic loci associated with PD risk through a meta-analysis of genome-wide association studies, suggesting that genetic susceptibility could modulate the effects of environmental exposures [27]. A study further supports the relationship between genetics and environmental factors by confirming that a genetic predisposition to higher PM_2.5_ levels increases the risk of AD and PD [134]. The genetic predisposition to PD is often associated with several key genes, including SNCA (alpha-synuclein), LRRK2 (Leucine-Rich Repeat Kinase 2), and GBA (glucocerebrosidase) [135]. This interplay between genetics and environmental exposures underscores the need for a personalized approach to public health interventions targeted at vulnerable populations, as genetic and environmental factors play crucial roles in neurological disease risk.

#### 4.4.2. Animal Studies

The primary pathology of PD involves the loss of dopaminergic neurons, particularly in the substantia nigra, accompanied by neuroinflammation [18]. Additionally, α-synuclein (a-syn) pathology and mitochondrial dysfunction are key contributors to PD pathogenesis [135]. Exposure to PM has been shown to increase the levels of proinflammatory factors (e.g., TNFα and IL-1β) and α-synuclein in the rat midbrain, suggesting that the midbrain is particularly susceptible to the neuroinflammatory effects of subchronic air pollution exposure [32]. PM exposure has also been linked to dopaminergic neuron loss in the substantia nigra [136]. Like other neurodegenerative diseases, oxidative stress is a significant mechanism in PD, contributing to the early degeneration of dopamine neurons [137]. Exposure to PM_2.5_ can exacerbate PD symptoms by increasing oxidative stress, reducing autophagy and mitophagy, and triggering mitochondria-mediated neuronal apoptosis [138]. In addition, chronic exposure to low doses of O_3_ has been shown to reduce the number of dopaminergic neurons, increase p53-immunoreactive cells, and elevate dopamine quinone levels due to oxidative stress [139]. These findings from animal studies closely mirror the neuropathological alterations observed in the brains of PD patients.

#### 4.4.3. Biological Mechanisms

Air pollution affects brain functions and contributes to the pathogenesis of PD through multiple pathways. Components of air pollution, such as PM_2.5_ and PAHs, can directly reach the brain, exerting neurotoxic effects and triggering neuroinflammation [140]. Additionally, air pollution may indirectly impact brain health by inducing systemic responses, such as releasing inflammatory cytokines or other chemical mediators from the lungs, which subsequently cause inflammation in the CNS [141]. Elevated levels of proinflammatory cytokines, including IL-1β, IL-2, IL-6, TNF-α, and C-reactive protein, have been observed in the blood of PD patients, potentially contributing to an increased risk of developing PD [142]. Airborne pollutants can also accumulate in the gastrointestinal tract, disrupting gut mucosal integrity, causing inflammation, and increasing intestinal permeability [143]. All of these may lead to the local accumulation of α-synuclein (α-syn) and alterations in the gut microbiome [144]. α-Syn is known to play a significant role in the pathogenesis of PD [135]. Its misfolded soluble oligomeric forms, known as protofibrils, are toxic entities that disrupt cellular homeostasis and induce neuronal death by affecting various intracellular processes, including synaptic function [145]. α-Syn can propagate to the brainstem via the vagus nerve and result in dopaminergic neuron loss [146], responsible for the characteristic motor symptoms associated with PD [24].

Furthermore, microbiome alterations induced by air pollutants may contribute to systemic inflammation, the release of neuroactive molecules, and neuroinflammation, further exacerbating PD pathology [147]. For example, exposure to ambient PM_2.5_ in mice resulted in significant alterations in gut microbial diversity, including a significant increase in Bacteroidales, which contributes to mucus layer degradation and an increase in gut permeability [148]. Additionally, the depletion of Lactobacillus, a strain critical for maintaining gut homeostasis, was observed in the treated group [148]. Together, these findings align with reports of an altered microbiota in PD patients [149], further implicating gut dysbiosis in the pathogenesis of PD.

Similarly, it is important not to neglect the lung–brain axis in our mechanistic understanding of neurological disease pathology. Recent studies suggest that impaired lung function or lung diseases (like COPD and asthma) due to air pollution are associated with declining cognitive ability and increased dementia risk by chronic hypoxemia [150,151]. Chronic hypoxemia can trigger systemic inflammation, oxidative stress, physiologic stress, cerebral arterial stiffness, and small-vessel damage [150,151]. Additionally, a lack of oxygen could contribute to hypoperfusion–reperfusion injury at the blood–brain barrier, leading to inflammation of the CNS [152,153]. This inflammation can be one of the contributing factors in the cognitive decline seen in vascular dementia [152,153]. The recent COVID-19 pandemic has highlighted another form of air pollution, emphasizing the lungs as a crucial component of our health and underscoring the importance of the lung–brain axis.

## 5. Discussion

As the growing body of research solidifies the implications of air pollution in neurological disease, it is important to mention that certain populations may be more susceptible to these harmful effects than others. Air pollution can pose a significant risk depending on demographics such as age and socioeconomic status. This puts children, the elderly, and low-income groups at heightened susceptibility to the neurological diseases associated with environmental toxins. Children are in critical stages of development, and their developing brains can be adversely affected by pollutants, leading to cognitive impairments and behavioral issues [3]. The elderly, often dealing with pre-existing health conditions, may also be less resilient to the adverse neurological effects of air pollution, resulting in heightened incidences of dementia and neurodegenerative diseases [19]. Low-income communities tend to reside in areas with higher pollution levels due to proximity to industrial facilities and traffic congestion, thus increasing their exposure to harmful pollutants and amplifying the risks of neurological impairment [154]. Research has also shown that marginalized communities are exposed to higher TRAP levels than the general population [155]. Moreover, research found that 96% of populations of the largest cities globally are exposed to PM_2.5_ levels that exceed the WHO guidelines [156], putting them at a greater risk of health concerns. Collectively, these vulnerable groups demonstrate a critical need for targeted public health initiatives to mitigate the impact of air pollution on health risks, specifically neurological health.

## 6. Conclusions and Future Directions

This review of the current literature provides a solid foundation for concluding that air pollutants are a significant environmental risk factor in the development of neurological diseases such as ASD, ADHD, AD, and PD. Both epidemiological and animal studies have been instrumental in establishing the association between air pollution and these diseases. The existing research has identified a strong positive association between increased exposure to air pollutants, such as PMs, NO_2_, and DEPs, and a higher risk of developing ASD. Similarly, compelling evidence suggests that air pollutants such as PM_2.5_, NO_2_, and CO are involved in the development of ADHD. Furthermore, research increasingly identifies air pollution, particularly PM_2.5_ and O_3_, as critical risk factors for the development of AD and PD. These findings are supported by animal and human studies, highlighting the neurotoxic effects of air pollutants and their complicated interactions with genetic and gender-related factors.

Despite the valuable insight these studies provide, the variability in results highlights the necessity for more high-quality studies. Several limitations hinder our understanding of the connection between these disorders and air pollution. For instance, methodological differences in exposure protocols in animal research can lead to inconsistent findings. While animal studies can provide valuable insights into neurotoxicity mechanisms, they often fail to fully replicate human pathology. More research is required to determine the appropriate dosages and routes of exposure that accurately reflect human exposure levels [5]. Similarly, in human studies, variations in methods for quantifying exposure can also lead to inconsistencies in results [5]. In addition, potential confounders, such as socioeconomic factors and pre-existing health conditions, can skew the results, making it challenging to attribute neurological disorders solely to air pollution exposure [24]. In addition, we acknowledge that the studies discussed in this review were conducted in diverse geographic locations with varying pollution levels, which further complicates comparisons. While the existing research established a preliminary relationship between air pollution and neurological disorders, the evidence to conclusively determine a causal relationship between air pollution and neurological diseases is still insufficient.

Another limitation of the existing research is the incomplete understanding of the etiology of these disorders. Without a comprehensive understanding of the development of these disorders, it is challenging to assess the role of air pollution in their pathogenesis. A deeper understanding of the mechanisms would help researchers investigate the effects and the underlying mechanisms of air pollution on neurological diseases. A deeper understanding of the neurotoxic effects of air pollution will enable researchers to focus on developing more adaptive care strategies, with a greater emphasis on preventive measures. These factors create a complex landscape that necessitates further investigation to fully elucidate the interactions between air pollution and neurological diseases in the future. Future research should employ in vitro and in vivo models to explore the pathways activated by air pollution exposure, including oxidative stress, neuroinflammation, and microglial activation. Future research should also include longitudinal studies that monitor populations over extended periods and can provide insights into how chronic exposure to air pollution affects cognitive decline and the onset of neurodegenerative diseases. Such studies could help identify vulnerable populations and critical exposure windows. By delving into these areas, we can better understand the connections between air pollution and neurodegenerative conditions, potentially leading to the development of preventive measures and adaptive care strategies.

## Data Availability

Data sharing does not apply to this article. All data are contained within this manuscript.

## Supplementary Materials

The following supporting information can be downloaded at: https://www.mdpi.com/article/10.3390/toxics13030207/s1, Table S1: Studies investigating air pollution in neurodegenerative disorders.

## Abbreviations

The following abbreviations are used in this manuscript:

ASD	Autism spectrum disorder
ADHD	Attention deficit hyperactivity disorder
AD	Alzheimer’s disease
PD	Parkinson’s disease
PM	Particulate matter
PM_2.5_	Particulate matter with diameter < 2.5 μm
PM_10_	Particulate matter with diameter < 10 μm
NO	Nitrogen oxide
NO_2_	Nitrogen dioxide
CO	Carbon monoxide
SO_2_	Sulfur dioxide
O_3_	Ozone
DEPs	Diesel exhaust particles
DEE	Diesel engine exhaust
VOCs	Volatile organic compounds
CNS	Central nervous system
NIH	National Institutes of Health
BBB	Blood–brain barrier
TNF-ɑ	Tumor Necrosis Factor-alpha
IL-1β	Interleukin-1 beta
IL-6	Interleukin-6
ROS	Reactive oxygen species
PAHs	Polycyclic aromatic hydrocarbons
GABA	Gamma-aminobutyric acid
BDNF	Brain-derived neurotrophic factor
Nrf2	Nuclear factor erythroid 2-related factor 2
MAPK	Mitogen-activated protein kinase
EC/BC	Elemental carbon/black carbon
SO_4_^2−^	Sulfate
NO_3_^−^	Nitrate
TBARs	Thiobarbituric acid reactive substances
MDA	Malondialdehyde
WHO	World Health Organization
TRAP	Traffic-related air pollution
H_2_O_2_	Hydrogen peroxide
GSH	Glutathione
HO-1	Heme oxygenase 1
SOD	Superoxide dismutase
CAT	Catalase
