# Supplementary material for: The Effects of Air Pollution on Neurological Diseases: A Narrative Review on Causes and Mechanisms
Source: Toxics. 2025 Mar 13;13(3):207. doi: 10.3390/toxics13030207 (PMC11946816; doi:10.3390/toxics13030207)
Supplement: Supplementary file 1 [file toxics-13-00207-s001.zip › toxics-3499038-supplementary.pdf]

toxics

MDPI

The Effects of Air Pollution on Neurological Diseases: A Narrative Review on Causes and Mechanisms

Margaret Lane †, Eleise Oyster †, Yali Luo * and Hao Wang *

Table S1. Studies investigating air pollution in neurodegenerative disorders.

Disorder	Category	Toxicant	Study Design	Key Findings	Source
Autism spectrum disorder (ASD)	Air pollution	$PM_{2.5}$, NO_2, O_3, CO	Epidemiological study; case–control study	Traffic-related air pollution in gestational and early-life periods was associated with ASD.	[11]
		$PM_{2.5}$, PM_{10}, NO_2	Epidemiological study; case–control study	$PM_{2.5}$, PM_{10}, and NO_2 during pregnancy and the first year of life were associated with ASD.	[12]
		$PM_{2.5}$	Meta-analysis	Increases in $PM_{2.5}$ were associated with increased risk of ASD in newborns.	[9]
		$PM_{2.5}$	Meta-analysis	$PM_{2.5}$ leads to greater risk of developing ASD compared to PM_{10}, NO_x, and other pollutants.	[9]
		CO, NO_2, PM's	Epidemiological study; case–control study	Prenatal, time-of-birth, or first-year-of-life exposure to TRAP was associated with an ASD diagnosis.	[11,12]
		CO, NO_2, PM's	Animal model	TRAP exposure during development induced behavioral, biochemical, and morphological changes that present in ASD.	[7,8]
		PM's	Animal model	Exposed mice to ultrafine particle	[59]

 https://doi.org/10.3390/13030207

				concentrations of 700 to 1200 for four hours, four days a week. Found an increase in glutamate levels in both male and female mice, causing them to have excitatory/inhibitory imbalances relevant to ASD.	
		DEPs	Animal model	Prenatal and nursing exposure to DEPs appeared to impact development of the CNS. Mice were exposed to 1000 μg/m^3 using a 5500-watt single-cylinder diesel engine generator for 4 h/day, 5 days/week. Mice exhibited increased locomotor activity and elevated levels of self-grooming and rearing.	[10]
Attention deficit hyperactivity disorder (ADHD)	Prevalence		Prospective cohort study	The population of children and adolescents with ADHD worldwide in 2020 was 5%.	[15]
			Systematic review	Increased to an estimation of 8% of children and adolescents with ADHD worldwide in 2024.	[80]
	Air pollution	CO	Systematic review	Children exposed to CO either pre- or postnatally often present with conduct disorders and ADHD later in life.	[16]
		PM_{10}, NO_2	Epidemiological study; cross-sectional study	Positive associations between PM_{10} and NO_2 and ADHD.	[14]

		PM_{10}, NO_2	Epidemiological study	Positive associations between PM_{10} and NO_2 and ADHD.	[13]
		$PM_{2.5}$, NO_2	Prospective cohort study	Children exposed to highest quintile of NO_2 and $PM_{2.5}$ in childhood had a 1.70-fold and 1.63-fold, respectively, increased risk of ADHD when compared to children exposed to the lowest quintiles of these pollutants.	[15]
		$PM_{2.5}$, NO_2	Prospective cohort study	Exposure in early life to these toxicants could be linked to an increased risk of a child developing ADHD.	[15]
		PM_{10}	Epidemiological study; cross-sectional study	Of all investigated pollutants, found the strongest association between PM_{10} exposure and ADHD.	[14]
		PM_{10}, NO_2	Epidemiological study; semi-individual study	In Germany, researchers found an association between NO_2 and PM_{10} and ADHD diagnosis.	[17]
		PAHs	Epidemiological studies	Studies conducted in New York and Barcelona both found a correlation between PAHs and ADHD in children.	[83,84]
Neurodegenerative disorders	Air pollution	$PM_{2.5}$	Animal model	Exposure to low-level $PM_{2.5}$ could accelerate the development of neurodegenerative pathologies in subjects with hypertension.	[23]

Alzheimer's disease (AD)	Air pollution	$PM_{2.5}$	Meta-analysis	Exposure to $PM_{2.5}$, NO_2, and O_3 is significantly associated with increased risk of AD.	[5]
		O_3	Systematic review	O_3 exposure contributes to AD pathology, exacerbating cognitive decline and amyloid deposition.	[40]
		$PM_{2.5}$	Systematic review	Exposure to $PM_{2.5}$, NO_2, and O_3 significantly increases the risk of AD, emphasizing environmental risks.	[19]
		$PM_{2.5}$	Meta-analysis	Each 10 μg/m^3 increase in $PM_{2.5}$ corresponds to a pooled hazard ratio of 4.82 for AD.	[105]
		O_3	Epidemiological cohort study	Ground-level O_3 exposure is directly linked to higher rates of cognitive decline among AD participants.	[109]
		PM_{10}, O_3	Longitudinal study	Long-term exposure to PM_{10} and ozone is significantly associated with an increased risk of AD.	[22]
		$PM_{2.5}$	Animal study	Low-level $PM_{2.5}$ inhalation increases neuroinflammation and synaptic alterations.	[23]
		$PM_{2.5}$	Cohort study	Increased exposure to $PM_{2.5}$ correlates with faster cognitive decline and increased Alzheimer's disease-related amyloidosis.	[25]

		$PM_{2.5}$	Animal study	Mice exposed to $PM_{2.5}$ show accelerated Alzheimer's disease-related pathology through increased amyloid plaque deposition.	[112]
		TRAP	Animal study	Chronic exposure to TRAP worsens disease markers in male and female rats genetically predisposed to AD.	[6]
Parkinson's disease (PD)	Air pollution	O_3, PM	Epidemiological study	Significant positive association between PD and both ozone and PM in North Carolina farmers.	[20]
		$PM_{2.5}$	Cohort study	Residents with higher $PM_{2.5}$-Mn concentrations exhibited worse motor function and cognitive performance.	[21]
		Ozone	Animal study	Chronic ozone exposure decreases the number of dopaminergic neurons in the substantia nigra.	[139]
